# Rate of Nutrition-Related Chronic Diseases Among a Multi-Ethnic Group of Uninsured Adults

**DOI:** 10.7759/cureus.28802

**Published:** 2022-09-05

**Authors:** Sahar Ajabshir, Sarah Stumbar, Innah Lachica, Kevin Gates, Zafar Qureshi, Fatma Huffman

**Affiliations:** 1 Cellular Biology and Pharmacology, Florida International University, Herbert Wertheim College of Medicine, Miami, USA; 2 Family Medicine, Herbert Wertheim College of Medicine, Miami, USA; 3 Herbert Wertheim College of Medicine, Florida International University, Herbert Wertheim College of Medicine, Miami, USA; 4 Obstetrics and Gynecology, Florida International University, Herbert Wertheim College of Medicine, Miami, USA; 5 Primary Care, Private Practice, Miami, USA; 6 Dietetics and Nutrition, Florida International University, Miami, USA

**Keywords:** access to healthcare and health outcomes of vulnerable populations, nutrition assessment, relationship between diseases and nutrition, obesity and nutrition, diabetes and nutrition, uninsured patients, health disparities, health disparities and vulnerable populations, health care disparities

## Abstract

The prevalence of nutrition-related chronic diseases, such as obesity, cardiovascular disease, and type 2 diabetes, among adults in the U.S. is of increasing importance. These conditions adversely affect the overall public health, health care systems, and economy. Marginalized minority groups have been disproportionally affected by these conditions. Lack of or inadequate health insurance limits access to health care, which contributes to poor health outcomes among individuals with these conditions. South Florida is home to diverse racial/ethnic minority groups, many of whom are uninsured and do not have access to expert-delivered nutrition education services. It is imperative to thoroughly study the health needs of these underserved patient populations and examine the rate of nutrition-related conditions among them in order to develop medically and culturally tailored nutrition education programs for them. Therefore, the aim of this study was to assess the prevalence of nutrition-related diseases among multi-racial/ethnic uninsured individuals living in South Florida. A four-week electronic health record of adult patients (N=272) from a free clinic in South Florida was analyzed. Spearman`s correlation and binary regression models were used to assess the relationship between the variables. The sample included females (65%) and males (35%). The mean age was 49.08±14.56 years. Overall, 87% had at least one nutrition-related condition, with overweight/obesity being the most observed (75.2%), followed by hypertension (39%), dyslipidemia (27.2%), and diabetes (23.9%). BMI was a significant predictor of the prevalence of hypertension among Whites (p=0.008) and Blacks (p=0.002) but not Asians (p=0.536). Overall, a high rate of nutrition-related chronic diseases was found among uninsured adults in this study. This supports the need for increased medically, culturally, and economically tailored nutrition education programs in free clinic settings.

## Introduction

Adults in the United States experience a high burden of nutrition-related diseases; with more than 70% being overweight/obese, 50% having diabetes/prediabetes, and 50% having cardiovascular disorders [1-4]. These chronic conditions adversely affect the overall public health, health care systems, and economy [1,5,6]. While their annual health and economic costs already surpass $1 trillion [6], the prevalence of nutrition-related chronic diseases is projected to increase significantly by 2030, suggesting an increasing burden on the U.S. economy [7,8]. Poor metabolic health, defined as the presence of hyperglycemia, dyslipidemia, hypertension, and abdominal obesity, has been associated with an increased risk of developing diabetes, cardiovascular, and other chronic diseases [9]. It is estimated that 88% of adults in the U.S. do not meet the optimal metabolic health targets [9]. Although metabolic abnormalities are multifactorial in origin, excess body fat can play a major role in their pathogenesis. Obesity may put individuals at an increased risk for the development of impaired immune function, local and systemic inflammation, chronic conditions, and all-cause mortality [10-14]. In addition, obesity has been associated with increased susceptibility to viral/bacterial/fungal infections and sub-optimal response to vaccination in the elderly [15].

While obesity is a genetically and physiologically complex condition, several factors, such as race/ethnicity, sociodemographic factors, dietary behaviors, and physical inactivity have been associated with the high prevalence of obesity among U.S. adults [16]. According to the Centers for Disease Control and Prevention (CDC), in 2018, the prevalence of obesity was highest among non-Hispanic Blacks (49.6%) followed by Hispanics (44.8%) [16]. The higher prevalence of obesity among these groups has been linked to increased incidence of other chronic diseases such as type 2 diabetes, cardiovascular disease, and certain cancers [17-19]. Nutritional status is a critical factor associated with an increased prevalence of obesity and obesity-related chronic conditions [20,21]. Nutritional status and diet quality may be affected by socioeconomic status, educational level, culture, acculturation level, food environment, and food security [20-24]. A cross-sectional study of 33,932 adults in the U.S. revealed significant disparities in diet quality based on race, socioeconomic status, and education [22]. Another study showed suboptimal achievement of dietary intake goals among male Hispanics and those with lower education levels and higher acculturation [23]. It is suggested that acculturation and duration of exposure to the U.S. environment may serve as critical risk factors for obesity among U.S.-born and foreign-born minority groups [25,26]. Clearly, nutrition influences individuals’ well-being, even more so for those from minority groups and disadvantaged backgrounds.

Structural inequalities among minority groups, such as income and wealth gaps, have been associated with decreased access to health care and the widening of health disparities [27]. Historically, structural inequalities have served as risk factors for increased transmission of infectious diseases and increased morbidity and mortality among underserved or medically disadvantaged populations [28]. According to the U.S. Bureau of Labor's statistics, women, Hispanics, and Blacks have a higher likelihood of being among low-wage earners [29]. Lack of or inadequate health insurance limits access to health care and nutrition education services, which contributes to poor health outcomes among low-income minority groups. In addition, socioeconomic disadvantages may lead to food and nutrition insecurity, which, in turn, can hinder the management of nutrition-related chronic diseases. These socioeconomic challenges have been highlighted during the global COVID-19 pandemic, as food insecurity becomes more widespread and sedentary lifestyle mandated, leading to poor nutrition and increased incidence of nutrition-related diseases.

South Florida is home to diverse racial/ethnic minority groups, many of whom are uninsured [30] and do not have access to expert-delivered nutrition education services. It is imperative to thoroughly study the health needs of these understudied and underserved patient populations and examine the rate of nutrition-related conditions among them in order to develop medically and culturally tailored nutrition education programs for them. Therefore, the aim of this study was to assess the prevalence of nutrition-related diseases among multi-racial/ethnic uninsured individuals living in South Florida.

## Materials and methods

A retrospective study of de-identified data of electronic health records from a free volunteer-staffed community clinic in South Florida, which provides primary care services to low-income, uninsured individuals was conducted. The study was approved by the Florida International University Institutional Review Board (reference number 108765). A four-week dataset was retrieved in order to provide a snapshot of patients seen in the clinic during normal operations. The data collection and analysis were both conducted in 2020. The dataset was de-identified based on the 18 elements of protected health information (PHI), and providers' information was also excluded. All males and females aged >18 years with at least one clinical encounter during this period were included. The dataset included patients' demographic information, including age, gender, race, ethnicity, and marital status; anthropometric measures, including height, weight, and BMI; vital signs; and diagnosed health conditions recorded as International Classification of Diseases (ICD)-10 codes.

Nutrition-related ICD-10 codes were identified using a guide for common diagnoses related to nutrition services by the Academy of Nutrition and Dietetics [31]. This list is inclusive of 153 ICD-10 codes under 16 categories for which registered dietitian nutritionists mostly receive referrals. The main categories include diabetes; kidney disease; weight management; diseases of the circulatory system; endocrine, nutritional, and metabolic diseases; diseases of the genitourinary system; mental, behavioral, and neurodevelopment disorders; diseases of the digestive system; pregnancy; diseases of the blood; diseases of the musculoskeletal system, diseases of the nervous system; symptoms, signs, and abnormal clinical and laboratory findings; certain infectious diseases; adult malnutrition; and no specific diagnosis (for general dietary counseling and surveillance).

For patients with more than one ICD-10 code related to a particular nutrition-related condition, the frequency of the condition was recorded only once. The patient's BMI was utilized for the identification of weight management conditions, including underweight, overweight, and obesity, as these conditions were not consistently recorded as a diagnosis in the initial dataset. The World Health Organization cut-off points were used for the BMI classification: underweight BMI <18.49 kg/m²; normal weight BMI = 18.5-24.99 kg/m²; overweight BMI = 25-29.99 kg/m²; obesity class I BMI = 30-34.99 kg/m²; obesity class II BMI = 35-39.99 kg/m²; and obesity class III BMI >40 kg/m². The incidence of each nutrition-related condition and the total number of nutrition-related conditions for each patient were determined based on the ICD-10 data and BMI classifications.

Descriptive statistical analysis was used for sample characteristics. Numerical and categorical data were compared using the independent t-test and chi-squared test, respectively. To assess the association between BMI, hypertension, and type 2 diabetes status, Spearman`s correlation analysis was performed. Binary regression models were used to assess the relationship between BMI and the incidence of hypertension, dyslipidemia, and type 2 diabetes. All analyses were two-sided with significance set at p<0.05. IBM SPSS version 26 (IBM Corp., Armonk, NY) was used to conduct statistical analysis.

## Results

Data from N=272 adults were included in the analysis, including females (n=177) and males (n=95). The characteristics of the sample are shown in Table 1. The mean age was 49.08±14.56 years with 12.5% of subjects being 65 years and older. The sample was composed of 26.5% Whites, 33.8% Blacks, 35.6% Asians/Asian-Indians, and 1.1% American Indians. Eight patients (3%) did not provide racial/ethnic information. About 24% were Hispanic/Latino. Among those with BMI values available (n=250), the mean BMI was 29.45±6.76 kg/m². More than 75% were overweight/obese (BMI >25 kg/m²), 22.8% had normal weight (BMI: 18.5-24.99 kg/m²), and 2% were underweight (BMI <18.49 kg/m²). The mean BMI among Whites, Blacks, and Asians/Asian-Indians were 29.2±7.09, 31.38±7.15, and 27.75±6.12 kg/m², respectively. One-way analysis of variance (ANOVA) followed by a Tukey post hoc test revealed a significantly higher BMI among Blacks compared to Asians/Asian-Indians [F(4,245)=3.69, p=0.002] (Table 2).

**Table 1:** Characteristics of the sample ¹BMI: Body mass index

Characteristic	Frequency (N=272)	Percentage (%)
Age		
18-64	238	87.5
≥65	34	12.5
Gender		
Female	177	65.1
Male	95	34.9
Race		
White	72	26.5
Black/African-American	92	33.8
Asian/Asian-Indian	97	35.5
American-Indian/Alaska Native	3	1.1
Not reported	8	2.9
Ethnicity		
Hispanic or Latino	65	23.9
Not Hispanic or Latino	200	73.5
BMI¹		
≤18.49	5	1.8
18.5-24.99	57	21
25-29.99	90	33.1
30-34.99	57	21
35-39.99	21	7.7
≥40	20	7.4
Not reported	22	8.1
Number of diagnosed nutrition-related conditions		
n=0	33	12.1
n=1	98	36
n=2	57	21
n=3	45	16.5
n=4	38	14
n=5	1	0.4
Hypertension		
Yes	105	38.6
Dyslipidemia		
Yes	74	27.2
Type 2 Diabetes		
Yes	65	23.9

**Table 2:** Inter-racial BMI comparisons (n=250) One-way analysis of variance followed by a Tukey post hoc test revealed a significantly higher BMI among Blacks compared to Asians/Asian-Indians [F(4,245)=3.69, p=0.002]. *p<0.05 was considered significant.

Reference Race	Compared race	P value
White	Black or African-American	0.27
	American-Indian and Alaska Native	1.00
	Asian and Asian-Indian	0.53
	Not reported	0.93
Black or African-American	White	0.27
	American-Indian and Alaska Native	1.00
	Asian and Asian-Indian	0.002*
	Not reported	1.00
Asian and Asian-Indian	White	0.53
	Black or African-American	0.002*
	American-Indian and Alaska Native	0.94
	Not reported	0.57

Of the 272 patients, 87.1% had at least one nutrition-related condition; 51.9% had two or more nutrition-related conditions; and 30.9% had more than three nutrition-related conditions (Table 1). Weight-related conditions were found to be the most common, observed in 77.2% of subjects (underweight 2%, overweight 36%, and obese 39.2%), followed by hypertension (38.6%), dyslipidemia (27.2%), and type 2 diabetes (23.9%). The prevalence of nutrition-related conditions was higher among the geriatric group (94.1%) compared to adults aged 18-65 years (87%) (Table 3). Gender-based analysis showed no significant differences in age and BMI among men and women. However, the rate of nutrition-related diseases was higher among men (91.6% men vs. 85.9% women). In addition, rates of hypertension (42% men vs. 36% women), dyslipidemia (30% men vs. 25% women), and type 2 diabetes (29.5% men vs. 20.9% women) were higher among men. The inter-racial group analysis showed the overall rates of nutrition-related conditions among Whites, Blacks, and Asians/Asian-Indians were at 84.7%, 88%, and 88.7%, respectively. This rate was at 84.6% among Hispanics/Latinos and 89% among non-Hispanics/Latinos. The rate of hypertension was highest among African-Americans (47.8%), followed by Whites (34.7%) and Asians/Asian-Indians (33%). The incidence of dyslipidemia was highest among Asians/Asian-Americans (32%), followed by African-Americans (26.1%) and Whites (25%). The incidence of type 2 diabetes was highest among Asians/Asians/Indians (33%), followed by African-Americans (21.7%) and Whites (15.3%) (Table 4).

**Table 3:** Frequency of the number of nutrition-related diseases based on age groups The overall rate of nutrition-related conditions was higher among the geriatric group compared to adults aged 18-65 years (94.1% versus 87%).

Number of diagnosed nutrition-related diseases	18-64 years (n=238)				≥ 65 years (n=34)			
	Frequency	%	Cumulative %	Frequency		%	Cumulative %	
0	31	13.0	-	2		5.9	-	
1	87	36.6	36.6	11		32.4	32.4	
2	46	19.3	55.9	11		32.4	64.7	
3	45	18.9	74.8	0		0	64.8	
4	28	11.8	86.6	10		29.4	94.1	
5	1	0.4	87.0	0		0	94.1	

There was a significant correlation between age and BMI (p=0.032, r=0.13). Binary regression analysis showed BMI is a good predictor of the incidence of hypertension (p<0.001). The association remained significant after controlling for covariates, including age, gender, race, and ethnicity (p<0.001). The inter-racial analysis revealed that BMI was a significant predictor of incidence of hypertension among Whites (p=0.008) and Blacks (p=0.002) but not Asians/Asian-Indians (p=0.536) in this group. No significant association was detected between BMI and incidence of dyslipidemia (p=0.064) and type 2 diabetes (p=0.181). However, the inter-racial analysis showed a significant association between BMI and incidence of type 2 diabetes among Whites (p=0.031). Inter-ethnic analysis showed a significant association between BMI and incidence of hypertension among both Hispanics/Latinos and non-Hispanic/Latinos (p=0.001 and p=0.003, respectively). BMI was a significant predictor of incidence of dyslipidemia (p=0.027) and type 2 diabetes (p=0.006) among Hispanics/Latinos but not non-Hispanic/Latinos. The model revealed age was a significant predictor of the incidence of hypertension (p<0.001), dyslipidemia (p<0.001), and type 2 diabetes (p<0.001) among all racial/ethnic groups. Bivariate analysis showed a 70.8% co-incidence of hypertension and a 64.4% co-incidence of dyslipidemia among adults with type 2 diabetes. The co-incidence of hypertension among adults with dyslipidemia was 73%.

**Table 4:** Rate of hypertension, dyslipidemia, and type 2 diabetes among different racial groups *The inter-racial analysis revealed that BMI is a significant predictor of incidence of hypertension among Whites (p=0.008) and Blacks (p=0.002), but not Asians/Asian-Indians (p=0.536). The analysis also showed a significant association between BMI and incidence of type 2 diabetes among Whites (p=0.031). p<0.05 was considered significant.

	BMI Mean ± SD			%Hypertension	%Dyslipidemia	%Type 2 Diabetes
Race						
White		29.2 ± 7.1		34.7*	25.0	15.3*
Black/African-American		31.4 ± 7.1		47.8*	26.1	21.7
American-Indian/Alaska Native		30.6 ± 8.6		33.3	0.0	0.0
Asian/Asian-Indian		27.6 ± 5.8		33.0	32.0	33.0
Not reported		31.2 ± 3.3		37.5	12.5	25.0

## Discussion

The primary aim of this study was to identify the prevalence of nutrition-related diseases among an uninsured group of patients in South Florida. Analysis of the data from 272 adult patients seen within four weeks pre-COVID-19 pandemic revealed that 87% had at least one nutrition-related condition; Overweight/obesity was the most observed condition in this group followed by hypertension, dyslipidemia, and type 2 diabetes. Given these chronic conditions are reportedly associated with increased morbidity and mortality due to COVID-19, the findings of this study highlight the need for tailored nutrition and diet interventions targeting this low-income, uninsured population.

Overall, more than 75% of patients were overweight/obese (BMI >25 kg/m²). The inter-racial analysis showed despite lower BMI levels among Asians/Asian-Indians, rates of dyslipidemia and type 2 diabetes were higher among them. This is consistent with previous studies suggesting Asians may be at higher risk of developing metabolic disorders at lower BMI values [32-38]. The results of the Nurses’ Health Study suggested a more pronounced effect of weight gain on the increased risk of developing type 2 diabetes among Asians. In fact, previous studies focusing on Asian and Asian-Indian populations noted that lower BMI cut-offs, classifying a BMI of 18.0-22.99 kg/m² as normal weight, a BMI of 23.0-24.99 kg/m² as overweight, and a BMI of >or=25kg/m² as obese, are optimal when looking at the prevalence of morbidity cases [34]. This may be due to body composition variations between people based on race, ethnicity, sex, and other factors such as physical activity and dietary intake [39-41]. Utilization of the same BMI cut-off points for all racial/ethnic groups in the clinical settings may result in underestimation of the cardio-metabolic risk among Asians.

Studies have indicated that alternative measures of obesity, such as waist circumference, may provide a more specific assessment of a person’s body fat deposition and therefore a more accurate prediction of cardio-metabolic diseases [42-45]. A study among 42,918 Chinese adults found waist circumference and waist-to-height ratio had greater associations with the incidence of diabetes compared to BMI [37]. Another study found a better predictor of body fat in Chinese adults incorporates age, height, skinfold thickness, and waist circumference [46]. These findings help explain why in the present study, the association between BMI alone, as a measure of body fat, was not as strong among Asians, compared to other racial/ethnic groups. Adjusting the BMI and waist circumference cut-off points based on the racial/ethnic background is a critical factor not only in assessing disease prevalence but also in developing prevention and treatment plans for nutrition-related diseases among Asian/Asian-Indian populations. Future multi-racial/ethnic studies may benefit from including additional measures of obesity, such as waist circumference and waist-to-hip ratio, for more accurate risk estimation of cardio-metabolic diseases in these populations.

The results of this study showed a significant association between BMI and the prevalence of dyslipidemia and type 2 diabetes among Hispanics. This may be due to several factors, including genetic predisposition and the small sample size. It is suggested that Hispanics/Latinos may be genetically predisposed to the development of insulin resistance, which then leads to the impairment of beta-cell functioning and glucose metabolism and thus results in type 2 diabetogenesis, especially among those with higher BMIs [47]. In addition to genetic predisposition, Hispanics/Latinos' unique microbiomes may be associated with an increased risk of developing obesity [48].

Our results showed a higher incidence of nutrition-related diseases among the geriatric group compared to adults aged 18-65 years. This suggests that older adults may face additional challenges in optimizing their nutritional health. For example, older adults are more likely to experience malnutrition due to food insecurity, chewing difficulty, and poor nutrient absorption [49,50]. Furthermore, an increased prevalence of micronutrient inadequacies has been reported among older adults with obesity, which may result in higher cardio-metabolic disease risk [51,52]. A recent study that examined the rate of food insecurity in relation to obesity and increased body fat composition found that food insecurity rose from 9% in 1999 to 18% in 2015 [53]. Food insecurity was strongly associated with poverty in the older adult population; and both food insecurity and obesity disproportionately affected minority populations, such as older African Americans and older Hispanics, compared to the general U.S. population [53,54]. Our findings confirm the results of this study and highlight the need for interventions tailored to address the nutritional needs of older adults.

We found higher rates of nutrition-related diseases, including hypertension, dyslipidemia, and type 2 diabetes, among men compared to women. This is consistent with other studies [55-57]. This difference in the rates of nutrition-related diseases between women and men can be a product of biological, sociocultural, and environmental influences [58,59]. It is suggested energy balance and metabolism are regulated differently among men and women, which can influence their susceptibility to certain metabolic disorders [57]. Differences in health-related behaviors among men and women can also contribute to their metabolic health outcomes. A recent study among 1066 adults in Taiwan found that women had healthier dietary habits, higher rates of physical activity, lower rates of smoking, and a lower incidence of obesity compared to men [60]. Another study on nutrition management found that Hispanic females with diabetes were more successful in achieving the recommended dietary cholesterol and sodium levels compared to Hispanic males with diabetes [23].

The present study had several strengths and limitations; to our knowledge, no previous studies have specifically explored the rates of nutrition-related chronic diseases among uninsured patients living in South Florida. This study shed light on the high rates of nutrition-related chronic diseases among underserved minority populations, thereby highlighting potential targets for future nutrition interventions in this community. Furthermore, the present study included individuals from diverse demographic backgrounds and was able to compare the rates of nutrition-related diseases among different age, gender, and racial-ethnic groups. Nevertheless, this study has several limitations; first, due to the retrospective nature of this study, there was a lack of consistent information on several variables such as the patient’s level of educational attainment, smoking status, and dietary behaviors. It is suggested that education level can be a determinant of healthy behaviors and thus plays a key role in nutritional intake and health outcomes. In addition, smoking can affect many of the chronic diseases studied in this dataset. The present study only used BMI to assess body fat status as waist circumference, a more accurate measure of abdominal obesity, was not measured routinely in the clinic when this study was conducted. In addition, BMI information was not available for 27 patients; however, they were not excluded from the study, as other nutrition-related diseases were recorded for them. Furthermore, specific information regarding patients' diets, how well their nutrition-related conditions were controlled, and their accessibility and usage of food assistance programs were not available.

## Conclusions

In conclusion, the results of this study showed a high rate of nutrition-related chronic diseases among uninsured adults from diverse racial/ethnic backgrounds living in South Florida. Obesity, hypertension, dyslipidemia, and diabetes were the most prevalent conditions in this group. In order to prevent these diseases and optimize treatment plans, nutrition programs tailored to patients of low socioeconomic status and culturally diverse diets must be incorporated within the free clinic settings.
